# Clustering and evolutionary analysis of small RNAs identify regulatory siRNA clusters induced under drought stress in rice

**DOI:** 10.1186/s12918-016-0355-3

**Published:** 2016-12-23

**Authors:** Inuk Jung, Hongryul Ahn, Seon-Ju Shin, Jukon Kim, Hawk-Bin Kwon, Woosuk Jung, Sun Kim

**Affiliations:** 1 Interdisciplinary Program in Bioinformatics, Seoul National University, Gwanak-Gu, Seoul, 151-747 Republic of Korea; 2 Department of Computer Science and Engineering, Seoul National University, Gwanak-Gu, Seoul, 151-747 Republic of Korea; 3 Department of Biomedical Sciences, Sunmoon University, Asan, 336-708 Republic of Korea; 4 Crop Biotechnology Institute, Green Bio Science & Technology, Seoul National University, Gangwon, Republic of Korea; 5 Department of Applied Bioscience, Konkuk University, Gwangjin-Gu, Seoul, Republic of Korea; 6 Bioinformatics Institute, Seoul National University, Gwanak-Gu, Seoul, 151-747 Republic of Korea

**Keywords:** SiRNA, Cluster, Dehydration, Rice

## Abstract

**Motivation:**

Drought tolerance is an important trait related to growth and yield in crop. Until now, drought related research has focused on coding genes. However, non-coding RNAs also respond significantly to environmental stimuli such as drought stress. Unfortunately, characterizing the role of siRNAs under drought stress is difficult since a large number of heterogenous siRNA species are expressed under drought stress and non-coding RNAs have very weak evolutionary conservation. Thus, to characterize the role of siRNAs, we need a well designed biological and bioinformatics strategy. In this paper, to characterize the function of siRNAs we developed and used a bioinformatics pipeline that includes a genomic-location based clustering technique and an evolutionary conservation tool.

**Results:**

By comparing the wild type Nipponbare and two drought resistant rice varities, we found that 21 nt and 24 nt siRNAs are significantly expressed in the three rice plants but at different time points under a short-term (0, 1, and 6 hrs) drought treatment. siRNAs were up-regulated in the wild type at an early stage while the up-regulation was delayed in the two drought tolerant plants. Genes targeted by up-regulated siRNAs were related to oxidation reduction and proteolysis, which are well known to be associated with water deficit phenotypes. More interestingly, we found that siRNAs were located in intronic regions as clusters and were of high evolutionary conservation among monocot grass plants. In summary, we show that siRNAs are important respondents to drought stress and regulate genes related to the drought tolerance in water deficit conditions.

**Electronic supplementary material:**

The online version of this article (doi:10.1186/s12918-016-0355-3) contains supplementary material, which is available to authorized users.

## Background

Plants are subject to long-term gradual water shortage in weeks or months, or to short-term water deficits in hours to days. Studies report that plants respond differently to long and short-term water deficit conditions. In long-term water shortage, the plant makes effort to optimize its use of resources and shortening their life cycle. In short-term water shortage, the plant minimizes water loss by shutting down respiratory procedures and triggers mechanisms to protect itself from damaging effects, e.g., oxidation that leads to proteolysis causing the break down of cells.

Traditionally, drought related research focus on coding genes. Recently, siRNAs in plants are being studied with great interest. Currently, siRNAs are classified into two primary categories by their type of precursors: hpRNAs whose precursor is single stranded forming a hairpin structure and siRNAs whose precursor is double stranded [1]. It is known that 21 and 24 nt length siRNAs are most abundant and are shown to have positive correlation with their host genes’ expression level under normal conditions [2]. The 21 and 24 nt siRNAs are known to be processed by the DICER-LIKE 4 in plants [3]. For their functional roles, studies report that siRNAs induce DNA methylation and chromatin remodeling [4], associate with repeats [5] and respond to environmental stimuli such as salt [6]. However, we still have little knowledge on the functional roles of siRNAs on coding genes in condition specific environments, especially drought stress. To characterize the gene regulatory roles of siRNAs in rice under drought conditions, we generated mRNA and small RNA high throughput sequencing data sets from three rice plants. The three plants are the drought susceptible wild type *Oryza sativa Nipponbare*, *WT* hereafter, the drought tolerant, AP2 over-expressed transgenic rice, *ap2* hereafter, and the naturally drought tolerant rice, Oryza sativa Indica Vandana. The *ap2* rice is derived from the WT by amplifying the *OsAP2* gene, where the *OsAP2* transgenic lines showed 20–35% increase in drought tolerance over wildtype at the vegetative growth stage (Ju-Kon Kim, unpublished data). Unfortunately, characterizing the role of siRNAs under drought stress using the small RNA-seq is difficult since a large number of heterogenous siRNA species are expressed under drought stress and non-coding RNAs have very weak evolutionary conservation. Thus, to characterize the role of siRNAs, we developed and used a bioinformatics pipeline to characterize function of siRNA, using a genomic-location based clustering technique and an evolutionary conservation tool, PhastCons. By performing the analysis of mRNA and small RNA data using the pipeline, the following findings are reported in this paper.

1. siRNAs are induced under drought condition in response to drought in all three rice plants but at a later stage in the drought tolerant plants,
2. a large number of siRNAs are enriched forming clusters within gene bodies,
3. the expression level of siRNA clusters negatively correlate with their target genes expression level and
4. intronic siRNA cluster regions show significant evolutionary conservation among the monocot grass plants.

## Methods

**Fig. 1:**
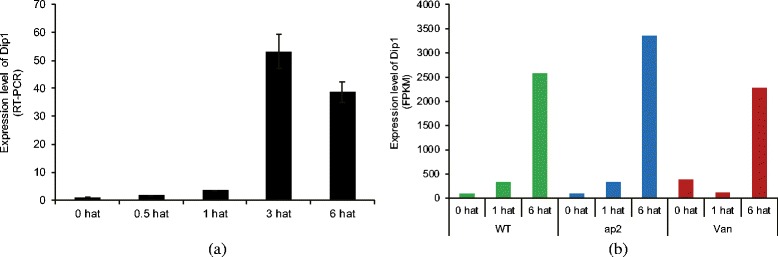
Expression level of DIP1 (Os02g0669100). **a** shows the expression level of DIP1 acquired by RT-PCR of WT only. **b** shows the expression level in units of FPKM (from high throughput data) for all plants. All three plants showed a significant increase in expression level at 6 hat, which is inline with the RT-PCR results

*Data* In this study, we conducted a short-term drought experiment with three time points, 0 hat (hour(s) after treatment), 1 hat, and 6 hat, on three different rice plants, WT, *ap2* and Vandana, generating mRNA and small RNA high throughput sequencing time-series data sets. The time points were chosen based on the expression level change of the drought induced gene (DIP1) measured by qRT-PCR as a metric of response to drought. Yi and colleagues [7] reported that DIP1 is induced upon drought treatment, reaching a peak at 2 hat and remaining constant until 8 hat, which could be used as a response marker for water deficiency. We also confirmed that three plants commonly showed response to drought at 6 hat by measuring the expression level of the DIP1 (Os02g0669100) gene (Fig. 1). The DIP1 was greately induced at 6 hat showing a clear response to drought. The time-series mRNA and small RNA transcriptome data of the three plants generated in this study are deposited in GEO under the accession number GSE74465.

**Fig. 2:**
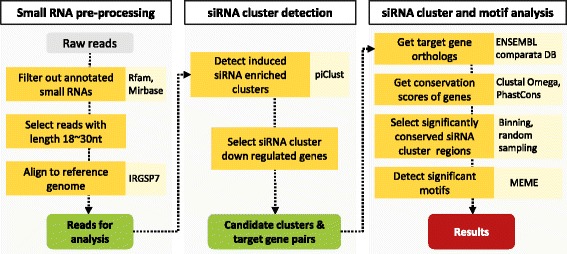
The analysis workflow consists of three parts with an objective to find siRNA clusters that down regulate genes and have high conservation. At each step, the tools or databases that were used are shown

To detect drought responsive siRNA clusters that show gene regulatory function and exhibit sequence conservation, a three step analysis procedure was designed for this study as illustrated in Fig. 2. The first step was for small RNA preprocessing. To collect siRNAs from the small RNA-seq data, reads that perfectly map to miRNAs, rRNA, tRNAs and snoRNAs annotated in miRBase [8] and Rfam [9] were filtered out. Furthermore, reads in size from 18 to 30 nt were selected and normalized by the total number of processed reads in each sample. Finally, the reads were mapped to the genome with two options, allowing no mismatch and up to five multiple mapping sites.

**Fig. 3:**
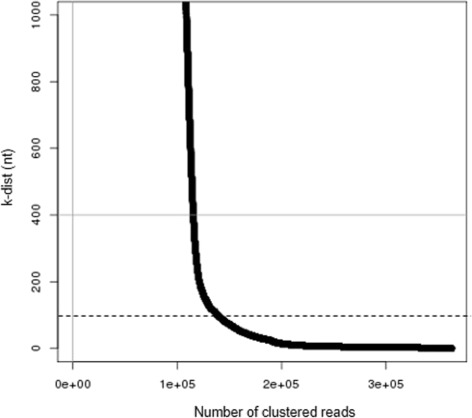
The *k*-dist analysis result of siRNA clusters using *k*=30. The optimal epsilon value (*black dashed line*) was observed at *k*-dist=100, which was set as the epsilon value. For robustness, we hav set minpts as 10

The second step was for genomic location-wise siRNA cluster detection and search for potential gene regulatory roles of siRNA clusters. Previous studies showed that siRNAs were present in siRNA enriched regions, referred to as phasiRNAs [10]. We also found that many genes harbor siRNA dense clusters within their gene body using a modified version of our previously implemented density based algorithm, piClust [11]. This clustering algorithm was used to eliminate noise in the data and efficiently detect highly reliable siRNA enriched clusters. piClust is based on the well known DBSCAN algorithm [12], which requires the configuration of two clustering parameters, epsilon and minpts. We configured the clustering parameters (i.e., epsilon=100nt, minpts=10) using the *k*-dist algorithm. For every mapped read, we observe the distance from a read to its *k* th nearest neighbor read which we refer to as *k*-dist. The *k*-dist is computed for every aligned read. Distance is measured in units of bp, starting from the left most loci of a read to the right. Once *k*-dist of each read is acquired, we sort the reads in an descending order based on their *k*-dist and plot it. In the plot, the user can observe a steep valley unless the *k*-dist follows a uniform distribution. The point at the valley of the *k*-dist is assigned to epsilon. Then *k* is automatically assigned to MinReads. As shown, the *k*-dist algorithm is a pre-step to configure the clustering parameters for piClust and details can be found in the original paper of DBSCAN. As a result, by performing *k*-dist with varying *k*s, we found that setting *k*=30 showed a robust result while retaining sufficiently many clusters. With *k* set as 30, the epsilon (i.e., the observed valley in the plot) was set as 100 with minpts set as 10. This configuration was calculated based on the WT plant at 0 hat and was applied to the other data sets since they did not show great variance. As an example, the *k*-dist analysis result of the WT plant at 0 hat is shown in Fig. 3.

With this parameter configuration, siRNA clusters with a minimum length of 100 nt with at least 10 siRNA transcripts are expected to be detected, which is a very strict criteria compared to other studies. In order to identify the expression correlation between siRNA clusters and their target genes, each cluster was quantified and associated to their target genes. Here, we define target genes as genes that embody one or multiple siRNA clusters. For multiple clusters that target a common gene, their expression levels were summed to compile a list of unique gene-siRNA cluster pairs.

The third step was to investigate further if genomic regions with overlapping siRNA clusters have conserved features. We did this by performing two bioinformatics analyses, genomic sequence conservation across multiple genomes and the motif analysis among siRNA cluster regions. To investigate on the evolutionary sequence conservation of siRNA clusters across five monocot grass plants, *Brachypodium distachyon*, *Oryza sativa*, *Setaria italic*, *Sorghum bicolor* and *Zea mays*, we measured the conservation strength of siRNA cluster regions. Since we are interested in gene regulatory siRNAs, siRNA clusters that have negative correlation with genes were selected as candidates. Orthologs of the selected genes targeted by siRNA clusters were searched for in the Plant Ensemble plant compara data [13]. Genes with orthologs in at least four plants, including *Oryza sativa*, were selected. Each gene and its orthologs were aligned using Clustal Omega [14] in order to acquire the PhastCons score [15]. The PhastCons scores were then binned with a bin size of 25 nt where each bin was assigned with the average PhastCons score of the bin. Bins were labeled as siRNA cluster bins and non-siRNA cluster bins depending on the presenece of an overlapping siRNA cluster. Per genomic feature, a t-test on the scores was performed between siRNA cluster bins and non-siRNA cluster bins for any significant difference (*p*≤0.05) in conservation. Since the number of siRNA cluster bins was fewer than non-siRNA cluster bins, the statistical test was repeated for 1000 sets of random selection of non-siRNA cluster bins to match the number of siRNA-cluster bins per feature. For the motif analysis, significantly conserved siRNA clusters were collected and used as input for motif discovery using MEME [16].

### Plant material and dehydration stress treatments

We germinated seeds of wild type rice (Oryza sativa Japponica Nipponbare) plants and Nipponebare-backgrounded *OsAP2* (Os02g0669100) over-expressed transgenic plants on petri-dishes with 200 mg/L Cefotaxime under 28, 16/8 h light/dark conditions for 5 days. We then cultured them for seedling in a 1.5 mL microfuge tube cellbox with Yoshida’s solution, under the same conditions for 4 days. After seedling, we moved them to an aquaculture container with Yoshida’s solution and cultured them under the same conditions for 8 days until the three-leaf stage. Afterward, we exposed them to drought stress conditions by removing the water in the aquaculture container. After a pilot experiment to determine the expression of DIP1 gene in the WT rice, we selected 0, 1, and 6 HATs as time points to measure omics data. The whole plants harvested at these time points were kept frozen in liquid nitrogen until DNA/RNA extraction.

### Measuring gene expression levels

To quantify gene expressions, the transcriptome RNA-seq data were first clipped and mapped to the Rice IRGSP1.0 reference genome [17, 18] using Tophat [19] and Bowtie [20]. Then the gene expressions (units in FPKM) were quantified using Cufflinks [21]. Genes not exceeding 1 FPKM at any time point were removed from the analysis data set.

## Results

### Delayed siRNA activation in drought resistant rice plants

**Fig. 4:**
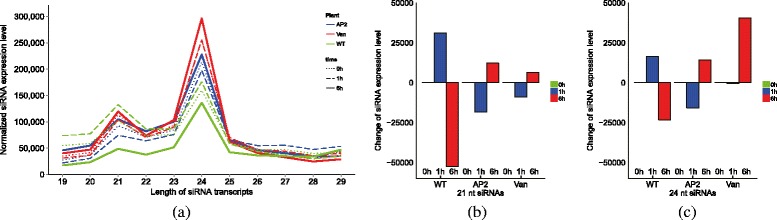
Expression patterns of siRNAs by length and time points. Sample size normalized expressions of siRNAs in each plant per time point. **a** The abundance of siRNAs per transcript length. The *blue*, *red* and *green colored lines* represent siRNA abundance in WT, *ap2* and Vandana plants respectively. The *dotted, dashed* and *solid line* types represent the time points 0, 1 and 6 h respectively. **b** The change of 21nt siRNAs expression level at each time point in respect to 0 h, and **c** the change of 24 nt siRNAs expression level at each time point in respect to 0 h (*0 h-green, 1 h-blue, 6 h-red*)

From the WT small RNA-seq data we obtained 7.9, 6.3, and 11 million reads at 0, 1 and 6 hat respectively. From *ap2*, we obtained 15.6, 11, and 9.3 million small RNA reads at 0, 1 and 6 hat respectively. From Vandana, we obtained 9.5, 10.3 and 11 million small reads at 0, 1 and 6 hat respectively. In all three plants and at all time points, two peaks were observed at 21 nt and 24 nt with the 24 nt reads being the most abundant one as shown in Fig. 4 a. Since the siRNA clusters are commonly observed to be up-regulated at least at one time point in all three plants, we find that the transcribed siRNAs may be a secondary effect of drought. However, the siRNA expression patterns were significantly different between WT and the drought resistance plants, *ap2* and Vandana (Fig. 4 b, c). In WT, siRNAs were mildly induced at 1 hat then decreased significantly at 6 hat. On the other hand, such expression pattern was the opposite in *ap2* where siRNA expression first decreased at 1 hat but then greatly increased at 6 hat. Vandana showed similar expression patterns to *ap2* only with greater abundance. Such expression patterns can be interpreted as: (1) up-regulation of siRNAs is a respondant signature to dehydration stress induced damage and (2) siRNA activation upon drought stress is delayed to a later stage, 6 hat, in drought resistant rice. This claim can be supported by the fact that siRNAs target genes are well known drought responsive genes (see “Discussion” section).

### siRNA clusters and their potential functional roles

From the siRNA clustering results the number of detected siRNA clusters differed among the three plants and time points. In WT, 6690, 6345 and 11128 siRNA clusters were detected genome wide in 0, 1 and 6 hat respectively. In *ap2*, 28429, 15715 and 19152 siRNA clusters were detected in 0, 1 and 6 hat respectively. In Vandana, 8799, 8816 and 14847 siRNA clusters were detected in 0, 1 and 6 hat respectively. Since a non-trivial number of clusters were plant and time specific, overlapping clusters were merged and collected for each plant. Furthermore, clusters residing in intergenic regions were filtered out. We found that 393, 781, and 488 genes were targeted by at least one siRNA cluster in WT, *ap2* and Vandana respectively.

**Fig. 5:**
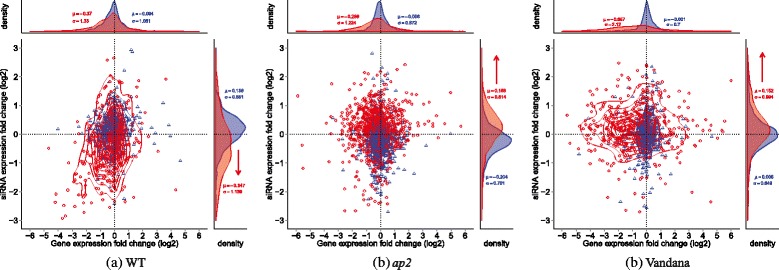
Correlation of siRNA cluster and gene expression levels. *Scatter plots* showing relation between siRNA cluster expression levels and the expression levels of their target genes. For each plant, the corresponding *scatter plot* is shown. The *x-axis* represents the log_2_ fold change of gene expressions, and the *y-axis* represents the log_2_ fold change of siRNA cluster expressions. The *blue triangles* indicate genes at 0-1 hat interval and the *red circles* indicate genes at 1-6 hat interval. Density plots of siRNA expression and gene expression are shown at the *top* and *right* of each scatter plot respectively

As shown in Fig. 5, during the 0-1 hat period, gene expression levels remained constant in *ap2* and Vandana. However, during the 1-6 hat period, most of these genes showed active regulatory patterns scattering along the horizontal axis with a bias towards the down regulation side. On the other hand, in WT, genes showed a more scattered formation mildly skewed to the down regulation side during 0-1 hat, which is coherent with the siRNA cluster expressions. Interestingly, during the 1-6 hat period, both genes and siRNAs showed strong down regulation, which pattern was not observed in *ap2* or Vandana. At the global scale, the pearsons correlation between gene and their targeting siRNA cluster expression level did not show significance for all three plants. However, it was observable that negative correlating pairs of siRNAs and their target genes are abundant compared to the others, especially in *ap2* and Vandana plants.

By performing gene set enrichment analysis of genes that are targeted by siRNAs, previously known drought responsive mechanisms, such as “Oxidation reduction-process (*P*-value= 3.88*e* ^−31^)” [22], “Small molecule metabolic process (*P*-value= 2.75*e* ^−25^)”, “Carbohydrate metabolic process (*P*-value= 2.83*e* ^−21^)” [23] and “Photosynthesis (*P*-value= 6.96*e* ^−14^)” [24], were found to be significantly enriched. The GO enrichment was performed using the PANTHER database (Release date: June 22^*nd*^ 2016). The full list of significantly enriched GO terms (178) are provided in the Additional file 1.

**Fig. 6:**
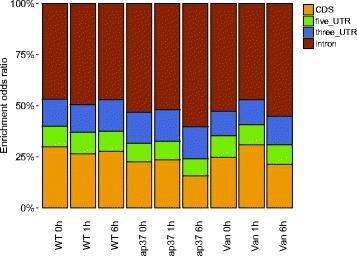
Location of siRNA clusters within gene bodies. Here, the enrichment odds ratio is shown per feature type. The ratio was normalized in respect to the length of each feature. In all time points and commonly across the three plants, the majority of siRNA clusters were located in intronic regions

**Fig. 7:**
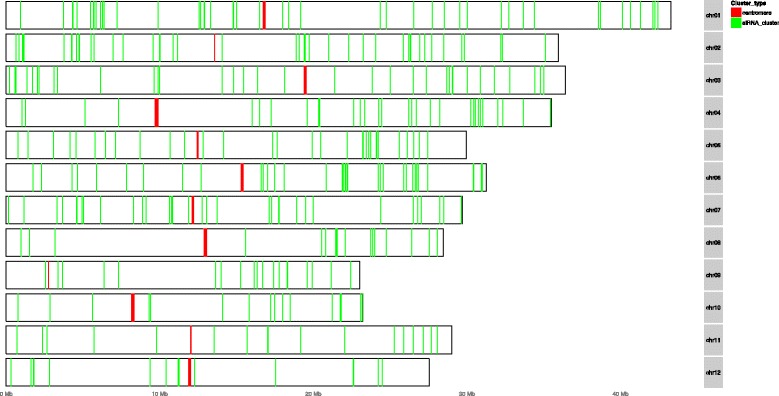
Genomic location of siRNA clusters. It was observed that the clusters were relatively densley located at telomeres. *Green stripes* represent the siRNA clusters, *red stripes* indicate the centromeres region in each chromosome

Among the detected siRNA clusters, we found that a majority, up to 50%, of siRNA clusters were located in intronic regions of their target genes in all three plants Fig. 6. The enrichment odds ratio was normalized by the length of features since UTR regions are expected to be much smaller than CDS or intron regions. Such siRNA enriched intronic regions were previously referred to as “sirtrons” [2]. The ratio of siRNA clusters did not vary significantly over time. From a genome wide perspective, we found that the siRNA clusters were located preferably near to telomeres rather than centromeres (Fig. 7), which was also observed in [25].

### Characterizing siRNA clusters using conservation and motif analysis

**Fig. 8:**
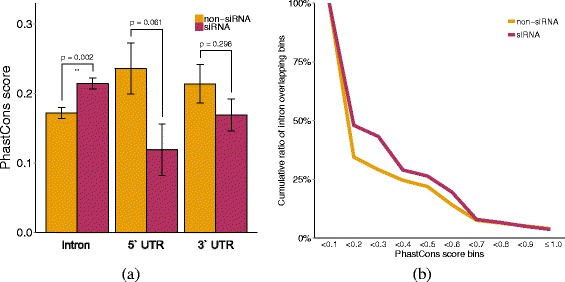
Conservation of siRNA cluster regions. Conservation comparison of PhastCons scores between siRNA cluster bins and non-siRNA cluster bins are shown. **a** The PhastCons score between siRNA cluster bins and non-siRNA cluster bins are compared per feature. Conservation of siRNA cluster regions were significantly high (*p*-value 0.002) in intron regions compared to intron regions with no overlapping siRNA clusters. **b** A cumulative graph of the ratio of intronic bins that belong to each PhastCons score range in steps of 0.1

For the investigation on the conservation of siRNAs, we excluded CDS regions since they yielded dominantly high conservation scores. Hence, only conservation scores along the 3 ^′^ UTR, 5 ^′^ UTR and intron regions were considered. We found that intronic siRNA cluster regions had significantly higher conservation compared to the intronic regions without siRNA clusters. The scores of 3 ^′^ UTR and 5 ^′^ UTR bins did not show statistical significance between siRNA cluster and non-siRNA cluster bins (Fig. 8). The significant conservation of intronic siRNA clusters across the monocot grass plants may imply the conservation of their regulatory roles.

**Fig. 9:**
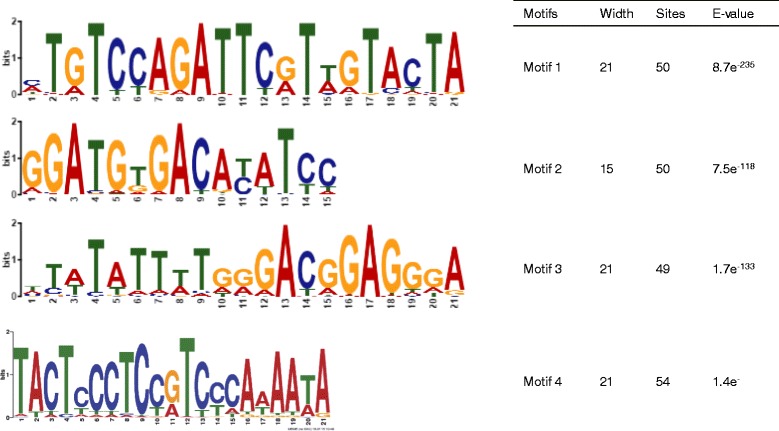
Detected motifs from siRNA clusters Four motifs were detected in the intronic siRNA cluster sequences

**Fig. 10:**
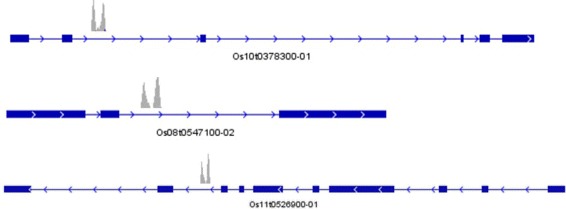
siRNA clusters with bimodal distribution within intronic regions. Here, three genes were selected to show several siRNA clusters with bimodal distribution characteristics. The *blue boxes* and *lines* represent rice transcripts exons and introns, respectively. The *gray spikes* represent the siRNA clusters that are located in a tandem structure. The *height* of the spikes represent the abundance

We further searched for specific sequence motifs from the intronic siRNA clusters using the MEME suit [26]. We first discovered motifs of length up to 51 nt using MEME [27]. Long motifs are difficult to interpret, hence, we repeated the motif search using MEME-ChIP [28]. From the motif analysis, using the sequences of the 364 intronic siRNA clusters as input, we found four sequence motifs that were present in up to 54 clusters with high significance as shown in Fig. 9. Three of the four clusters had length of 21 nt, whereas one had length of 15 nt. Interestingly, motif 1 and 2 were observed to be located in a tandem strucuture at opposite strands. Actually, many siRNA clusters showed bimodal enrichment at two sites close together. As an example, three genes that were targeted by such siRNA clusters are shown in Fig. 10. The gene harboring the motifs are listed in the Additional file 2. A majority of genes harboring an siRNA cluster or an motif have well known biological functions related to drought stress, such as the bZIP transcription factor, GA2-oxidase or Cytochrome P450. In summary, we found that siRNA cluster regions share common motif signatures and are conserved across the monocot grass plant, which suggests the potential regulatory role of siRNAs.

## Discussion

Studies related to drought tolerance have shown that drought tolerant cultivars have higher constitutive level of anti-oxidative enzymes compared to drought sensitive one [29–31]. In addition, drought tolerant cultivars have higher level of proteolysis in roots as well as shoots compared to the drought sensitive ones [32], while during the recovery from drought, proteolytic activity diminishes [33]. In [34] siRNAs are reported to be expressed in response to biotic and abiotic stress, such as high salt, temperature extremes, hypoxia, and immunity. However, there are no studies on the functional role of siRNAs in aspects of the drought tolerant mechanism. Here, we report, for the first time to the best of our knowledge, that siRNAs were induced in response to drought stress, but at a later stage in drought tolerant rice plants. Such differently expressed siRNAs have potentially regulatory roles on their target genes. Among the siRNA target genes, the gene set enrichment analysis results showed that oxidation reduction and photosynthesis related genes were differently regulated between WT and drought tolerant cultivars. While intron sequences are known to have low or no conservation, we found intronic regions with enriched overlapping siRNAs to have comparatively high conservation rates, especially in intronic regions (Fig. 8). Generally UTR regions are exptected to have higher conservation rate than introns. Compared to the 4258 intronic siRNA clusters, only 891 and 1677 siRNA clusters were detected in 5 ^′^ and 3 ^′^ UTR regions. Also shown in the results above, we found that siRNA clusters were majorly found within intronic regions. One possible explanation could be that UTR are less targeted since these regions are important in terms of biological mechanisms such as gene regulation. Another reason may be that siRNA clusters detected in the UTR region exhibit different characteristics to the intronic siRNA clusters. Due to this fact, we chose the intronic siRNA clusters for further characterization. By performing motif analysis, we found 4 motifs across 87 genes that were located within siRNA cluster regions. This was further supported by the motif analysis that showed that eight genes with all four motifs were Acyl-protein thioesterase 2, Ent-kaurene oxidase (Cytochrome P450), allergen-related, Subtilisin-like protease, TGF-beta receptor, type I/II extracellular region family protein, carboxylyase, Transcriptional factor B3 domain containing protein and NusB/RsmB/TIM44 domain containing protein.

## Conclusions

In summary, we report that conserved siRNAs are responders indicating the drought related damages to the plant as the drought stress continues and their functional roles are related to well known drought related pathways.

## Additional files

Additional file 1 The GO (Gene Ontology) enrichment results of genes with siRNA targeting sites. (XLSX 20 kb)

Additional file 2 The detected motif sequences in siRNA clusters located in intronic regions. (XLSX 98 kb)
